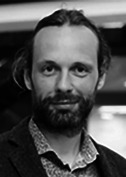# Synthetic Enzyme‐Catalyzed CO_2_ Fixation Reactions

**DOI:** 10.1002/cssc.202100159

**Published:** 2021-03-10

**Authors:** Godwin A. Aleku, George W. Roberts, Gabriel R. Titchiner, David Leys

**Affiliations:** ^1^ Department of Biochemistry University of Cambridge 80 Tennis Court Road Cambridge CB2 1GA UK; ^2^ Manchester Institute of Biotechnology Department of Chemistry University of Manchester 131 Princess Street Manchester M1 7DN UK

**Keywords:** biocatalysis, carboxylation, cascade reactions, CO_2_ fixation, enzymes

## Abstract

In recent years, (de)carboxylases that catalyze reversible (de)carboxylation have been targeted for application as carboxylation catalysts. This has led to the development of proof‐of‐concept (bio)synthetic CO_2_ fixation routes for chemical production. However, further progress towards industrial application has been hampered by the thermodynamic constraint that accompanies fixing CO_2_ to organic molecules. In this Review, biocatalytic carboxylation methods are discussed with emphases on the diverse strategies devised to alleviate the inherent thermodynamic constraints and their application in synthetic CO_2_‐fixation cascades.

## Graphical abstract

**The fix is in**: This Review examines progress made in developing (de)carboxylases for application in organic synthesis for CO_2_ fixing reactions. It highlights the synthetic scope of promising (de)carboxylase enzymes and the numerous strategies devised to increase enzymatic carboxylation yield, including application of (de)carboxylases in synthetic cascades.

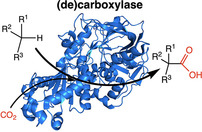

## 1 Introduction

Carbon dioxide is an inexpensive and renewable carbon source that can be harnessed as a building block to generate industrial chemicals, green fuels, pharmaceutical precursors, agrochemicals, and polymers.[^1^, ^2^, ^3^] Utilizing CO_2_ in this way reduces dependence on fossil fuels, promoting sustainable chemistry and anthropogenic CO_2_ recycling.^[4]^ The current climate change threat demands new strategies to mitigate excess atmospheric CO_2_, potentially by converting it into useful commodities. Several catalytic methodologies for the conversion of CO_2_ to economically viable fuels and chemicals have sought to develop cost‐effective strategies for either the reduction of CO_2_[^5^, ^6^] (e. g., to methane, methanol, or dimethyl ether) or the incorporation of CO_2_ into organic molecules.^[7]^ The latter route is otherwise referred to as synthetic CO_2_ fixation and represents an immensely attractive sustainable synthetic approach for the utilization of CO_2_ as renewable C1 building block.[^8^, ^9^, ^10^]

In practice however, efficient synthetic CO_2_‐fixation routes are rare, owing to both the thermodynamic barrier associated with fixing CO_2_ to organic compounds, as well as the high energy requirement for substrate activation. Recent efforts to address these limitations have led to the development of promising chemocatalytic strategies which have shown potential for industrial exploitation.^[8]^ Notably, the use of bases such as KO*t*Bu, Cs_2_CO_3_, aluminum‐based Lewis acids, and transition metals to promote C−H carboxylation have resulted in significant improvements with respect to synthetic scope, yields, and, in some cases, green credentials.[^9^, ^11^, ^12^, ^13^, ^14^] However, these chemocatalytic carboxylation routes are still associated with imperfect regioselectivity and harsh operating conditions, including elevated temperatures and pressures.

In contrast, biological CO_2_ fixation reactions are often highly selective and occur under mild conditions. Several functionally diverse families of carboxylases have evolved to catalyze physiologically important CO_2_‐fixing reactions, playing pivotal roles in the natural carbon cycle.^[16]^ A key enzyme in this regard is ribulose‐1,5‐bisphosphate carboxylase/oxygenase (RuBisCO) which catalyzes carboxylation of ribulose‐1,5‐bisphosphate (RuBP); a central transformation step in photosynthesis.[^17^, ^18^] The exciting chemistry performed by carboxylases, especially those occurring in autotrophic pathways (e. g., RuBisCO) have inspired chemists to develop novel biomimetic carboxylation reactions.[^11^, ^19^] However, attempts to exploit nature's abundant carboxylases by developing synthetic carboxylation routes have garnered slow progress, attributed to strict substrate specificity, poor catalytic efficiency and requirement of complex, expensive or unstable co‐factors.^[18]^ Nevertheless, some natural carboxylases have been investigated as in vitro and in vivo carboxylation catalysts for the synthesis of target compounds.[^20^, ^21^, ^22^, ^23^]

Recent focus has thus been targeted towards the development and application of (de)carboxylases which catalyze reversible decarboxylation in microbial biosynthetic and degradation pathways.[^16^, ^24^, ^25^, ^26^, ^27^] These enzymes frequently exhibit relaxed substrate tolerance, simple cofactor requirements, stability and inherent evolvability (e. g., towards broad substrate tolerance, stability, high catalytic efficiency, solvent tolerance and high regio‐ and stereoselectivity). Since the carboxylation process is often thermodynamically disfavored, the exploitation of decarboxylases as carboxylation catalysts must be supported by effective strategies to overcome the thermodynamic constraints associated with the uphill carboxylation reaction.

In this Review, we examine recent efforts aimed at developing synthetic biocatalytic carboxylation methods with emphasis on (i) proof of concept application of (de)carboxylases for one‐step biocatalytic synthesis of target carboxylic acids at analytical, semi‐preparative and preparative scales; (ii) diverse strategies devised to alleviate the inherent thermodynamic constraint of CO_2_ fixation, (iii) the exploitation of these enzymes to develop synthetic biocatalytic cascades that can provide access to carboxylic acids and their derivatives through CO_2_ fixation, and (iv) opportunity to expand the biocatalytic (de)carboxylation toolbox by exploiting and evolving prenylated flavin (prFMN)‐dependent decarboxylases as one of the promising routes for enzymatic carboxylation. This Review is not intended to be exhaustive, hence does not cover CO_2_‐fixation routes employing metabolic and genetic engineering approaches as these have been recently reviewed elsewhere.[^28^, ^29^] Similarly, the Review does not cover aspects relating to the biochemistry and physiological roles of (de)carboxylases which have previously been reviewed by our group and others.[^24^, ^25^, ^27^, ^30^]

## 2 An Overview of Synthetically Targeted (De)carboxylases

**Scheme 1:**
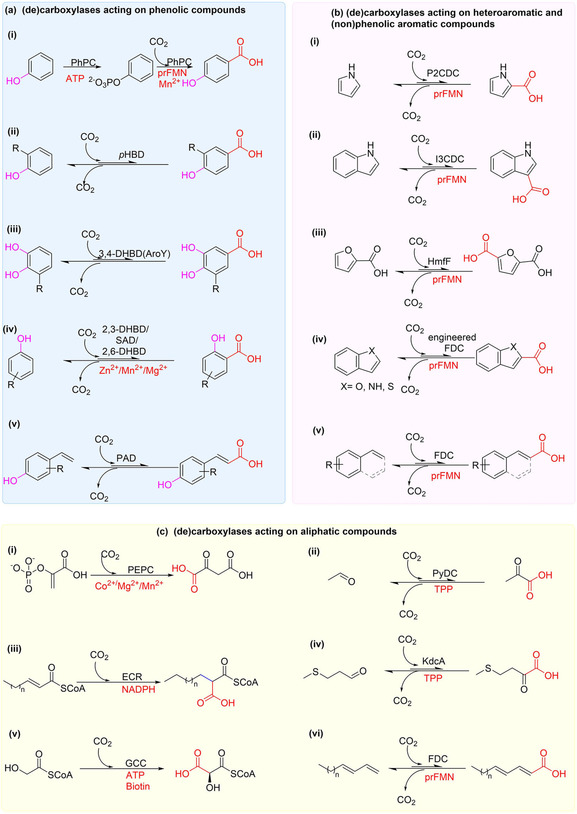
A selection of synthetically promising (de)carboxylases acting on distinct but diverse substrate groups and showing site of carboxylation: (a) acting on phenolic compounds; (b) acting on heteroaromatic and nonphenolic aromatic compounds; (c) acting on aliphatic compounds. Organic/divalent metal ions cofactors presented in red. Organic cofactors: ATP=adenosine triphosphate; prFMN=prenylated flavin; TPP=thiamine pyrophosphate; NADPH=nicotinamide adenine dinucleotide phosphate (reduced). Enzymes: PhPC=phenylphosphate carboxylase; *p*HBD=*para*‐hydroxybenzoic acid decarboxylase; DBHD=dihydroxybenzoate decarboxylase; PAD=phenolic acid decarboxylase; P2CDC=pyrrole‐2‐carboxylate decarboxylase; I3CDC=indole‐3‐carboxylate decarboxylase; HmfF=2,5‐furan dicarboxylic acid decarboxylase; FDC=(fungal) ferulic acid decarboxylases; PEPC=phosphoenolpyruvate carboxylase; pyruvate decarboxylases; KdcA=branched‐chain α‐ketoacid dehydrogenase; ECR=carboxylating enoyl‐thioester reductases; GCC=glycolyl‐CoA carboxylase.

A diverse panel of (de)carboxylases with distinct substrate specificities (Scheme 1), ranging from those acting on phenolic substrates (Scheme 1a), heteroaromatic and nonphenolic aromatic compounds (Scheme 1b) to (functionalized) aliphatic substrates (Scheme 1c) have been biochemically characterized by different groups. Given the high energy requirement for substrate (de)activation during the (de)carboxylation process, most (de)carboxylases employ an organic cofactor such as thiamine pyrophosphate (TPP), biotin, (prenylated) flavin, pyridoxal, and/or divalent metal ions including Zn^2+^, Mg^2+^, Mn^2+^, Fe^2+^ and Co^2+^.^[31]^

For example, thiamine pyrophosphate (TPP) is frequently employed by decarboxylases acting on (branched) α‐keto acids.^[32]^ Members of (TPP)‐dependent decarboxylases such as pyruvate decarboxylase (PyDC) and branched α‐keto acids decarboxylase (KdcA) have recently been investigated for application as carboxylation catalysts on oxyfunctionalized aliphatic compounds.[^33^, ^34^] Other aliphatic carboxylases such as NADPH‐dependent carboxylating enoyl‐thioester reductases (ECRs)[^21^, ^35^, ^36^] and the recently developed glycolyl‐CoA carboxylase (GCC) have been exploited towards the carboxylation of CoA thioester‐containing aliphatic substrates. The latter enzyme has been developed from a biotin‐dependent propionyl‐CoA carboxylase.[^37^, ^38^]

Another class of decarboxylases emerging as an attractive set of (de)carboxylation biocatalysts are the UbiD‐family of enzymes. These enzymes catalyze reversible (de)carboxylation using the newly discovered prenylated flavin (prFMN) cofactor,[^26^, ^30^, ^39^] and have shown promise for application as (de)carboxylation catalysts.[^40^, ^41^, ^42^, ^43^] One example is the prFMN‐dependent dihydroxybenzoic acid (de)carboxylase (AroY), which catalyzes the prFMN‐dependent *para*‐carboxylation of catechols,^[40]^ while another distinct class of prFMN‐dependent enzymes, fungal ferulic acid decarboxylases (FDCs) catalyze the prFMN‐mediated reversible (de)carboxylation of a broad range of acrylic acid derivatives.^[41]^ FDCs are a particularly exciting class of (de)carboxylases owing to their potential for carboxylation of nonactivated terminal alkenes and (hetero)aromatic compounds.

The versatile UbiD‐enzyme family also feature members that catalyze the regioselective (de)carboxylation of (hetero)aromatic compounds. These include; 2,5‐furandicarboxylic acid decarboxylase (hmfF),^[42]^ pyrrole‐2‐carboxylate decarboxylases (P2CDC),[^44^, ^45^] indole‐3‐carboxylate decarboxylase (I3CDC),^[46]^ and phenyl phosphate carboxylase (PhPC).^[47]^ Emerging evidence has shown/predicted that these enzymes employ the prFMN cofactor in their catalysis, in a similar fashion to characterized members of the UbiD enzyme family.^[39]^

Several decarboxylases employ divalent metal ions without requiring any organic cofactors. For example, 2,6‐dihydroxybenzoic acid decarboxylases (2,6‐DHBDs)^[48]^ and 2, 3‐DHBDs^[49]^ utilize Zn^2+^ and Mg^2+^/Mn^2+^ respectively to facilitate the carboxylation of dihydroxybenzenes, while the aliphatic carboxylase, phosphoenolpyruvate carboxylase (PEPC)[^50^, ^51^] employs either Co^2+^, Mg^2+^, or Mn^2+.^ in its carboxylation process.

Finally, while cofactors are often essential in most enzymatic (de)carboxylation reactions, a few families of decarboxylases operate without the use of any cofactors,^[31]^ relying on the active site architecture for catalysis. These enzymes use a general acid‐base mechanism, often involving substrate‐assisted catalysis, typically by the substrate phenolic moiety. Phenolic acid decarboxylases (PADs), which catalyze the direct carboxylation at the β‐carbon of the vinyl group of hydroxystyrenes,^[52]^ are a notable example of synthetically useful cofactor‐free decarboxylases.

## 3 One‐Step CO_2_ Fixation Routes for the Synthesis of Carboxylic Acids

### 3.1 Synthesis of *para*‐hydroxybenzoic acids through C−H carboxylation of phenols

**Scheme 2:**
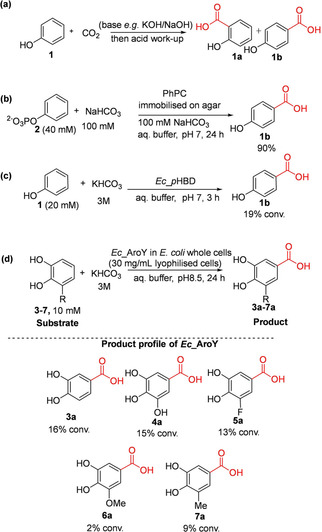
Regioselective *p*‐carboxylation of phenylphosphate, phenol and catechols catalyzed by reversible nonoxidative decarboxylases. (a) Kolbe‐Schmitt carboxylation method (b) Carboxylation of phenyl phosphate catalyzed by phenyl phosphate carboxylase (PhPC)^[55]^ (c) Biotransformation performed with phenol decarboxylase from *Enterobacter cloacae (Ec_p*HBD*)* ^[57]^ *(*d*)* Biotransformation performed using prFMN dependent 3,4‐dihydroxybenzoic acid decarboxylases (AroY) from *E. cloacae* (*Ec*_AroY).^[40]^

Following the discovery of (de)carboxylases in microbial aromatic degradation pathways,^[53]^ exploitation of these enzymes for sustainable carboxylation of aromatic compounds began to attract attention. Initial efforts were focused on developing efficient biocatalytic carboxylation methods that can potentially replace the well‐established Kolbe‐Schmitt approach for the carboxylation of phenol **1** (Scheme 2a).^[54]^ In the 1990s, the first promising biocatalytic alternative to Kolbe‐Schmitt approach was demonstrated by applying a Mn^2+^‐dependent phenyl phosphate carboxylase (PhPC), an enzyme involved in phenol metabolism. PhPC catalyzes regioselective *p*‐carboxylation of phenylphosphate **2** to yield *p*‐OH benzoic acid **1 b**,[^47^, ^55^] coupling carboxylation to dephosphorylation, likely dependent on prenylated flavin (prFMN) cofactor.^[39]^ By employing cell‐free extract containing PhPC or the partially purified enzyme supported on low melting agar, Aresta et al. achieved carboxylation of **2** to yield **1 b** affording up to 90 % yield (Scheme 2b).^[55]^ PhPC was also applied as a carboxylation catalyst under supercritical CO_2_ (ScCO_2_) conditions.^[56]^ Unfortunately, the PhPC system does not catalyze direct carboxylation of phenol **1**, as an ATP‐dependent phosphorylation step is required to activate phenol prior to carboxylation, limiting applicability.

Efforts were re‐directed towards identifying reversible (de)carboxylases capable of catalyzing direct *p*‐carboxylation of phenol, leading to the isolation and characterization of a cofactor‐free *p*‐hydroxybenzoate decarboxylase from *Enterobacter cloacae (Ec_p*HBD*)*.^[57]^ In the presence of 3 M KHCO_3_, the enzyme was shown to catalyze regioselective *p*‐carboxylation of **1** to yield **1 b**, affording conversion of 19 % (Scheme 2c).^[57]^ Yoshida et al. later characterized 3,4‐dihydroxybenzoate decarboxylase from *E. cloacae* (*Ec*_AroY). By employing *Ec*_AroY as the carboxylation catalyst and 3 M KHCO_3_ as CO_2_ source, regioselective *p*‐carboxylation of catechol **3** furnished the corresponding carboxylate **3 a** in 28 % conversion. (De)carboxylation activity of *Ec*_AroY was initially only observed with whole‐cell or cell free extract, whereas the isolated enzyme rapidly lost activity upon purification. Recent work by Payer et al. has revealed that AroYs belong to the UbiD‐like prFMN‐dependent enzyme family, and indeed, in vitro (de)carboxylase activity of *Ec*_AroY was restored following reconstitution with prFMN.^[40]^ To showcase the synthetic scope of AroYs, Payer et al. applied recombinant *E. coli* whole cells containing over‐expressed *Ec*_AroY with 3 M KHCO_3._ This revealed that catechol derivatives **3**–**7**, bearing electron‐withdrawing or ‐donating groups at the *meta* position relative to the carboxylation site, were converted, yielding the corresponding carboxylic acids **3 a**–**7 a** in low conversion values, up to 16 % (Scheme 2d). In contrast, catechol derivatives bearing *o*‐substituents or simple phenols were unreactive.^[40]^

### 3.2 Synthesis of *ortho*‐hydroxybenzoic acids by C−H carboxylation of phenols

Several dihydroxybenzoic acid decarboxylases (DHBDs) capable of catalyzing regioselective *o*‐carboxylation have been characterized and their synthetic applicability have been explored. For example, by employing *E. coli* cells expressing a cofactor‐free salicylic acid decarboxylase from *Trichosporon moniliiforme* (SAD_*Tm*) or its engineered variant, Kirimura and co‐workers performed a highly regioselective *o*‐carboxylation of phenol **1** and *m*‐aminophenol **8**, yielding salicylic acid **1 a** and *p*‐aminosalicylic acid **8 a** respectively.[^58^, ^59^] An impressive yield of up to 70 % carboxylation product was obtained from 100 mM *m*‐aminophenol, with a supply of 2 M KHCO_3_ as the CO_2_ source.^[59]^ Similarly, Nagasawa and co‐workers synthesized 2,6‐dihydroxybenzoic acid **10 a** by employing 2,6‐dihydroxybenzoic acid decarboxylase from *Agrobacterium tumefaciens* (2,6‐DHBD*_At)* catalyzing regioselective *o‐*carboxylation of 1,3‐dihydroxybenzene **10** with conversion of 30 %.^[60]^

**Scheme 3:**
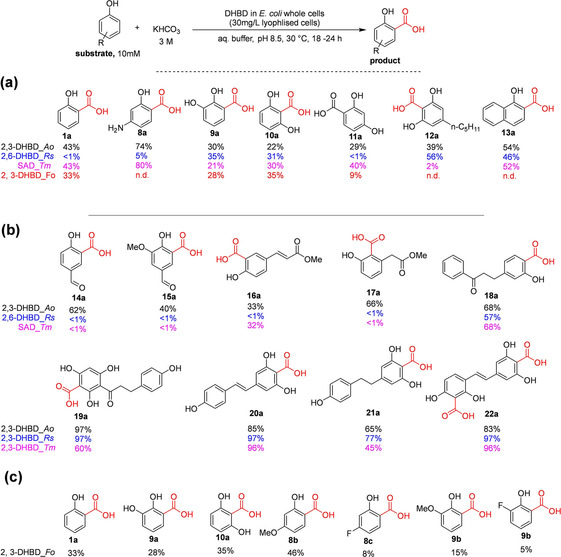
Examples of regioselective *ortho*‐carboxylation of phenolic compounds catalyzed by recombinant *E. coli* whole cells containing a 2,3‐dihydroxybenzoic acid decarboxylase (2,3‐DHBD) reported by (a) Wuensch et al.,^[61]^ (b) Plasch et al.,^[63]^ and (c) Zhang et al.^[64]^ 2,3‐DHBD_*Ao*=2,3‐dihydroxybenzoic acid decarboxylase from *Aspergillus oryzae*; 2,6‐DHBD_*Rs*=2,6‐dihydroxybenzoic acid decarboxylase from *Rhizobium* sp. and SAD_*Tm*=salicylic acid decarboxylase from *Trichosporon moniliiforme*. 2,3‐DHBD_*Fo*=2,3‐dihydroxybenzoic acid decarboxylase from *Fusarium oxysporum*. Conversion values (%) obtained with 2,3‐DHBD_*Ao*, 2,6‐DHBD_*Rs*, and SAD_*Tm* are given in black, blue, and pink, respectively.

Within the last decade, studies from Faber's group have highlighted the significant synthetic potential of nonoxidative decarboxylases for carboxylation of phenolic compounds. Wuensch et al. investigated the substrate tolerance and regioselectivity of three previously characterized bacterial DHBDs for the *o*‐carboxylation of structurally diverse simple phenols and dihydroxybenzene derivatives.^[61]^ Biotransformation reactions were performed using lyophilized recombinant *E. coli* whole cells expressing one of the following *ortho*‐decarboxylases: 2,3‐dihydroxybenzoic acid decarboxylase from *Aspergillus oryzae* (2,3‐DHBD_*Ao*),^[62]^ 2,6‐dihydroxybenzoic acid decarboxylase from *Rhizobium* sp. (2,6‐DHBD_*Rs*)^[60]^ and SAD_*Tm*.^[58]^ These enzymes display broad substrate tolerance enabling *o*‐carboxylation of several simple phenolic compounds, as well as dihydroxybenzene derivatives **1** and **8**–**13**. In all cases, the enzymes displayed excellent regioselectivity (Scheme 3a).^[61]^ Plasch et al. further demonstrated an extended substrate scope for these catalysts; several polyphenolic compounds were carboxylated with good to excellent conversions of up 97 %, enabling access to a wide range of *o*‐carboxylated (di)hydroxyaromatic carboxylic acids **14 a**–**22 a** (Scheme 3b).^[63]^ For example, preparative scale enzymatic carboxylation of resveratrol **20** was achieved, affording the carboxylated product **20 a** in excellent isolated yield of 95 %.^[63]^ Of the three enzymes, 2,3‐DHBD_*Ao* displayed the broadest substrate scope, accepting a wide range of phenolic compounds including those bearing other reactive groups such as carbonyls **14 a**, **15 a**, and **18 a**, as well as bulky polyphenolic compounds **18 a**–**22 a**.^[63]^

Later, Zhang et al. also characterized a 2,3‐DHBD from *Fusarium oxysporum* (2, 3‐DHBD_*Fo*) to further expand the biocatalytic toolbox for *o*‐carboxylation.^[64]^ This homologue exhibited tolerance for high substrate loading. Phenol, catechol and other substituted phenolics were carboxylated at saturated KHCO_3_ levels with conversion up to 46 % (**1 a**, **8 b**, **c**, **9 a**–**c**; Scheme 3c).^[64]^

### 3.3 One‐step CO_2_ fixation for the synthesis of heteroaromatic carboxylic acids

**Scheme 4:**
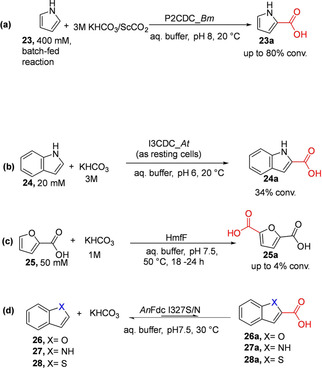
Regioselective carboxylation of heteroaromatics: (a) Carboxylation of pyrrole by pyrrole‐2‐carboxylate decarboxylase from *Bacillus megaterium* (P2CDC_*Bm*).^[44]^ (b) Carboxylation of indole by indole‐3‐carboxylate‐decarboxylase from *Arthrobacter nicotianae* (I3CDC_*At)*.^[46]^ (c) Carboxylation of furoic acid to yield 2,5‐furandicarboxylic acid (FDCA) catalyzed by reversible prFMN‐dependent decarboxylase HmfF^[42]^ (d) (de)carboxylation of benzo‐fused *O*‐, *N*‐, and *S*‐containing heteroaromatic carboxylic acids catalyzed by evolved *An*Fdc variants (I327S or I327N).^[43]^ *An*Fdc=*Aspergillus niger* ferulic acid decarboxylase.

In view of the prevalence of heteroaromatic carboxylic acids as structural component in pharmaceuticals, agrochemicals, and industrial chemicals; regioselective synthetic methodologies that enable access to these compounds under mild reaction conditions would be very desirable. Nagasawa and co‐workers exploited two heteroaromatic acid decarboxylases, pyrrole‐2‐carboxylate decarboxylase (P2CDC)[^44^, ^65^, ^66^] and indole‐3‐carboxylate decarboxylase (I3CDC)^[46]^ to demonstrate proof‐of‐concept regioselective C−H carboxylation of pyrrole and indole respectively. The P2CDC enzyme from *Bacillus megaterium (*P2CDC_*Bm)* catalyzed regioselective carboxylation of pyrrole **23** to pyrrole‐2‐carboxylic acid **23 a** with supply of saturated solution of KHCO_3_.[^65^, ^66^] Optimization of the P2CDC‐based process for carboxylation of pyrrole in a batch reactor enabled preparative scale reaction at 300 mM substrate loading and up to 80 % yield was achieved (Scheme 4a).^[65]^ Matsuda et al. further showed that whole cells of *B. megaterium* containing P2CDC_*Bm* can be adapted for carboxylation of **23** under ScCO_2_; affording 59 % yield after 1 h, a significant improvement when compared to conversion of 7 % obtained with reaction under atmospheric pressure. A continuous flow process employing the immobilized form of the biocatalyst in a flow reactor under 6.5 MPa CO_2_ pressure enables carboxylation of pyrrole with a yield of 24±7 μmol h^−1^; representing a 25‐fold improvement when compared to a corresponding batch process.^[67]^

Recently, Payne et al. characterized a P2CDC from *Pseudomonas aeruginosa (*P2CDC_*Pa* or PA0254/HudA).^[68]^ PA0254/HudA share 44 % sequence identity with P2CDC_*Bm* and catalyze a prFMN‐dependent reversible (de)carboxylation pyrrole‐2‐carboxylate **23** as well as related furan and thiophene analogues, albeit in much lower efficiency for the latter compounds.^[45]^ However, benzo‐fused heteroaromatic compounds such as indole‐2‐carboxylate and indole‐3‐carboxylate were rejected. Carboxylation biotransformation employing P2CDC_*Pa* (PA0254/HudA) as the catalyst and supplying 1 M KHCO_3_/1.5 MPa CO_2_ afforded up to 55 % conversion of **23** to **23 a**.^[45]^

Another distinct heteroaromatic decarboxylase, indole‐3‐carboxylate decarboxylase from *Arthrobacter nicotianae* (I3CDC‐*At*) was shown to catalyze the regioselective carboxylation of indole **24** to furnish indole‐3‐carboxylic acid **24 a**; conversion of 34 % was achieved when 3 M KHCO_3_ was supplied as the CO_2_ source (Scheme 4b).^[46]^

Emerging data on the characterization of prFMN‐dependent enzymes and phylogenetic analysis suggest P2CDCs and the I3CDC_*At* belong to the UbiD enzyme family and employ prFMN‐mediated (de)carboxylation,[^39^, ^45^] although this remains to be verified for I3CDC.

The Leys group have further exploited prFMN catalysis to significantly expand the product profile for heteroaromatic carboxylic acids. For example, Payne et al. reported the carboxylation of furoic acid **25** to the corresponding dicarboxylic acid (2,5‐furandicarboxylic acid (FDCA, **25 a**)) using purified thermotolerant *Pelotomaculum thermopropionicum* HmfF (*Pt*HmfF) and 1 M bicarbonate, affording conversion of up to about 4 % (Scheme 4c).^[42]^ Compound **25 a** was also generated under gaseous CO_2_ at 32 bar pressure albeit at a significantly lower conversion when compared to reaction employing elevated bicarbonate concentration as the CO_2_ source. A substrate profiling study monitoring decarboxylation by HPLC or by H/D exchange reveals that *Pt*HmfF also displayed tolerance towards pyrrole‐2‐carboxylate **23 a**, and oxazole‐2‐carboxylic acid as substrates but not the thiophene analogue. In addition, an activating carboxylic acid group is required as the corresponding unsubstituted heteroaromatics are nonreactive. Hence, like P2CDCs and I3CDC_*At*, the synthetic scope of HmfF is limited.

Recently, structure‐guided protein engineering was used to evolve *Aspergillus niger* ferulic acid decarboxylase (*An*FDC) into a broad spectrum heteroaromatic (de)carboxylase (*An*Fdc I327S/N) catalyzing reversible decarboxylation of oxygen‐, nitrogen‐, and sulfur‐containing benzo‐fused heteroaromatic carboxylic acids **26 a**–**28 a** (Scheme 4d).^[43]^ The *An*Fdc I327S variant was exploited as the carboxylation catalyst for the C−H functionalization of benzofuran **26** to the corresponding carboxylate derivatives through cascade biocatalysis.^[43]^

### 3.4 Synthesis of α,β‐unsaturated carboxylic acids by C−H carboxylation of (hydroxy)styrenes

**Scheme 5:**
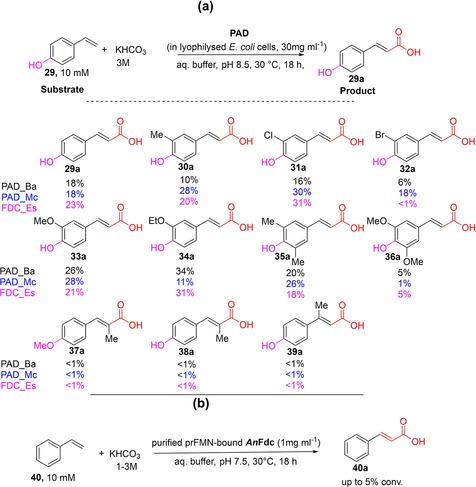
Regioselective β‐carboxylation of (*p*‐hydroxy)styrenes (a) Regioselective β‐carboxylation of *para*‐hydroxystyrenes by applying *E. coli* whole cells expressing co‐factor free phenolic acid decarboxylases (PADs)/ferulic acid decarboxylase (FDC) from bacterial sources.^[52]^ Enzymes: Phenolic acid decarboxylase (PAD) from *Bacillus amyloliquefaciens* (PAD*_Ba*); from *Mycobacterium colombiense* (PAD_*Mc*), and ferulic acid decarboxylase (FDC) from *Enterobacter* sp. (FDC_*Es*). For clarity, conversion values (%) obtained with PAD_*Ba*, PAD_*Mc* and FDC_*Es* have been given in black, blue, and pink, respectively. (b) Regioselective β‐carboxylation of styrene catalyzed by prFMN‐bound ferulic acid decarboxylase from *Aspergillus niger* (*An*Fdc).^[43]^

Faber and co‐workers developed a novel synthetic method for β‐carboxylation of *p*‐hydroxystyrene derivatives. Bacterial phenolic acid decarboxylases (PADs) were shown to catalyze the direct carboxylation of the vinyl group (sp^2^C−H) of hydroxystyrenes in the presence of KHCO_3_ as co‐substrate.[^52^, ^61^] PADs displayed excellent regio‐ and stereoselectivities by acting exclusively on the β‐carbon of the alkene moiety of hydroxystyrenes, generating (*E*)‐*p*‐coumaric acid derivatives. Application of recombinant *E. coli* whole cells expressing novel and previously characterized bacterial PADs[^52^, ^61^, ^69^] exhibited regioselective β‐carboxylation of a wide variety of *p*‐hydroxystyrene derivatives **29**–**36** in the presence of 3 M KHCO_3_, yielding the corresponding carboxylic acids **29 a**–**36 a** in low to moderate conversions (Scheme 5).^[52]^ Related substrates bearing methyl substitution at the α‐carbon or β‐carbon to the carboxylic group (as in **37 a**–**39 a**) were unreactive, likely due to the steric constraint posed by the substituent.

**Scheme 6:**
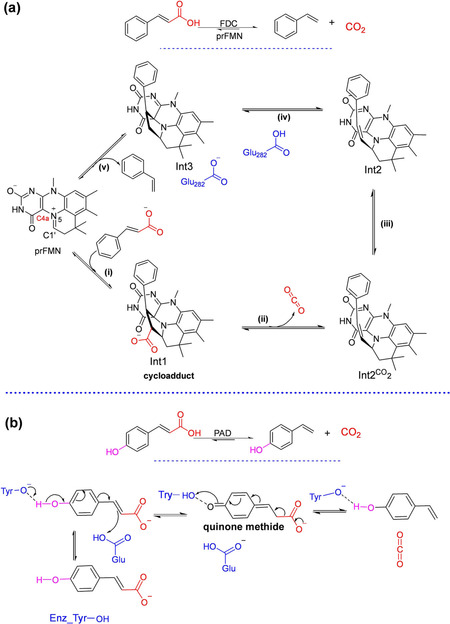
Mechanism of FDC‐catalyzed (de)carboxylation of phenylacrylic acids (e. g., cinnamic acid) *vs* PAD‐mediated (de)carboxylation of phenolic analogues (e. g., *p‐*coumaric acid). (a) Mechanism for decarboxylation of cinnamic acid by covalent catalysis using the prFMN iminium involving an initial 1,3‐dipolar cycloaddition between the dipolarophile of the substrate and the azomethine ylide‐like species of prFMN^iminium^ resulting in a cycloadduct species Int1. The Glu282 side chain mediates the protonation step to form Int3.[^70^, ^71^] The decarboxylation product styrene is released through a cycloelimination process. (i–v)=1,3‐dipolar cycloaddition, decarboxylation, CO_2_ to Glu 282 exchange, protonation and cycloelimination, respectively. (b) A general acid‐base mechanism employed by phenolic acid decarboxylases (PADs) and enabled by an essential phenolic moiety.[^52^, ^72^]

In agreement with the substrate‐assisted mechanism of PAD (Scheme 6), related compounds lacking the *p*‐hydroxy group (e. g., styrene/cinnamic acid) or the vinyl moiety (e. g., phenol) did not react. Indeed, PAD‐mediated (de)carboxylation relies on simple acid‐base catalysis,^[12]^ rendering a *para*‐hydroxy phenolic functional group obligatory for activity. This severely limits the scope of application of bacterial PADs. In contrast, fungal ferulic acid decarboxylases (FDCs) can act on ‘nonphenolics’ by virtue of a distinct prFMN‐dependent mechanism (Scheme 6).

Substrate profiling studies have established that a phenolic moiety is not essential for FDC‐prFMN‐mediated (de)carboxylation activity. Indeed, recent work has shown that several acrylic acid derivatives bearing plain phenyl, substituted aromatic, heteroaromatic as well as aliphatic groups were efficiently decarboxylated by the prFMN‐dependent fungal FDCs to provide access to structurally diverse terminal alkenes in up to 99 % conversion.[^41^, ^73^]

However, the synthetic utility of fungal FDCs as carboxylation catalysts is limited by the severe thermodynamic constraint of this reaction, prFMN‐bound *An*Fdc‐or its variant from *Saccharomyces cerevisiae* (*Sc*Fdc)‐catalyze the carboxylation of styrene in the presence of 1–3 M KHCO_3_ or NH_4_HCO_3_ to form cinnamic acid, affording conversion of up to 5 % (Scheme 5b). The reversibility of the FDC‐catalyzed carboxylation inspired the development of novel enzymatic CO_2_‐fixation cascades for the C−H *β*‐carboxylation of nonactivated styrene applying *An*Fdc/ScFdc as the carboxylation catalysts^[43]^ (see Section 5).

### 3.5 Synthesis of short‐chain aliphatic carboxylic acids

In primary metabolism, several distinct families of (de)carboxylases catalyze the (de)carboxylation of oxy‐functionalized aliphatic C2−C6 intermediates such as: acetate, glycolate, glyoxalate, oxalate, pyruvate, malonate, succinate, fumarate, malate and oxaloacetate to mention a few. In some interesting cases, these pathways feature tandem carboxylation‐decarboxylation steps catalyzed by a carboxylase‐decarboxylase pair. For example, biotin and ATP‐dependent acetyl CoA carboxylase and β‐keto acyl synthase catalyze tandem carboxylation‐decarboxylation reactions in fatty acid biosynthesis,^[24]^ whilst in gluconeogenesis tandem carboxylation/decarboxylation reactions are mediated by ATP and biotin‐dependent pyruvate carboxylase and PEP carboxykinase (PEPCK), catalyzing carboxylation of pyruvate to oxaloacetate and the subsequent decarboxylation and phosphorylation respectively. Although originally thought to perform irreversible decarboxylation, studies of the *Bacillus subtilis* isozyme of PEPCK has shown that in the absence of the enzyme pyruvate kinase, PEPCK can perform the reverse reaction, albeit in a highly limited fashion.^[74]^ In some plants, PEPCK homologues catalyze decarboxylation of oxaloacetate in the Hatch‐Slack pathway of C_4_ carbon fixation,^[75]^ or the Crassulacean acid metabolism (CAM) pathway.[^75^, ^76^] Whereas PEP carboxylase (PEPC) catalyzes the opposing reaction to PEPCK, the carboxylation and dephosphorylation of PEP to form oxaloacetate.^[50]^

From a biotechnological application viewpoint, these (de)carboxylases have been largely explored to optimize microbial biosynthetic pathways to improve production titers of the native pyruvate‐based compounds through genetic manipulation and synthetic biology approaches.[^16^, ^22^, ^23^, ^77^, ^78^, ^79^] However, there are limited reports of biocatalytic exploitation of these aliphatic carboxylic acid (de)carboxylases to develop one‐step carboxylation methods for the preparation of short‐chain carboxylic acids and derivatives.

#### 3.5.1 One‐step synthesis of α‐keto acids and aliphatic dicarboxylic acids

**Scheme 7:**
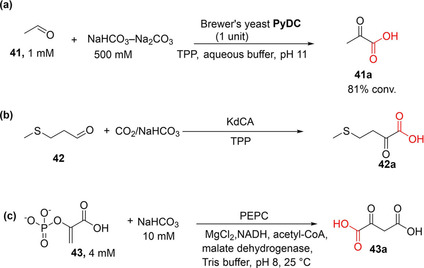
One‐step CO_2_‐fixation transformations for oxy‐functionalized aliphatic compounds. (a) Carboxylation of acetaldehyde to yield pyruvic acid with CO_2_, catalyzed by pyruvate decarboxylase.^[33]^ (b) One‐step CO_2_‐fixation route to the corresponding α‐keto acid, although, owing to severe thermodynamic limitation of this reaction, significant carboxylation was only achieved when the carboxylate was removed from the equilibrium by further enzymatic derivatization.^[34]^ (c) Conversion of phosphoenolpyruvate+CO_2_ into oxaloacetate catalyzed by phosphoenolpyruvate carboxylase (PEPC).[^80^, ^81^]

Miyazaki et al. demonstrated the synthetic utility of the thiamine pyrophosphate (TPP)‐dependent pyruvate decarboxylase (PyDC) as carboxylation catalyst to convert acetaldehyde **41** and CO_2_ to pyruvic acid **41 a**. Following optimization of reaction conditions, particularly pH and bicarbonate concentration, the PyDC‐catalyzed carboxylation of **41** afforded **41 a** in up to 81 % conversion (at pH 11 and 500 mM bicarbonate as a CO_2_ source; Scheme 7a).^[33]^

Other aliphatic acid decarboxylases that have recently been investigated include the TPP‐dependent branched‐chain α‐keto acid decarboxylase (KdcA) from the Ehrlich pathway. Skerra and co‐workers showed that KdcA can be applied as carboxylation catalyst to synthesize L‐methionine from the precursor aldehyde **42** and gaseous CO_2_ at 2 bar pressure through KdCA‐catalyzed carboxylation (to **42 a**; Scheme 7b), linked to an enzymatic reductive amination. The amination step was incorporated to alleviate the effect of the unfavorable chemical equilibrium of the CO_2_ fixation step.^[34]^

Chang et al. also demonstrated the use of PEPC for the in vitro carboxylation of phosphoenolpyruvate (PEP) **43** to generate oxaloacetate **43 a** (Scheme 7c). It was reported that the conversion value was improved when carbonic anhydrase (CA) was incorporated to increase the availability of CO_2_ (hydrated form, HCO_3_^−^) in the reaction buffer.^[80]^ Del Prete et al. utilized a similar approach, applying a thermostable PEPC and CA for the conversion of PEP and CO_2_ to produce oxaloacetate even at 60 °C.^[81]^

#### 3.5.2 Carboxylation of CoA thioesters

**Scheme 8:**
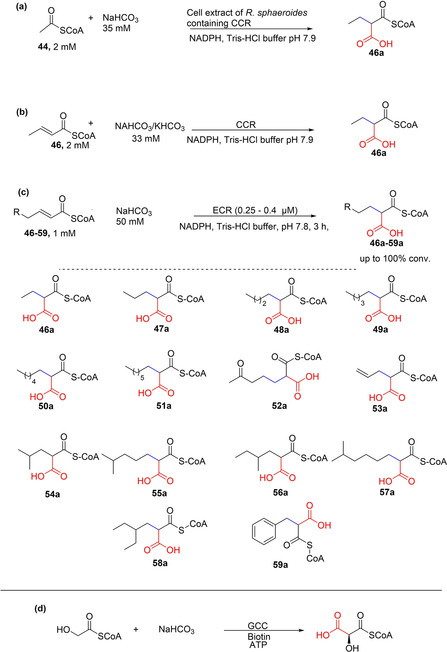
Enzymatic carboxylation of coenzyme A (CoA) thioesters. (a) Conversion of acetyl CoA into ethylmalonyl CoA through a multistep reaction involving a carboxylation step, catalyzed by a Rhodobacter sphaeroides *lysate*.^[82]^ (b) Carboxylation of propionyl CoA catalyzed by crotonyl CoA carboxylase/reductase (CCR)^[82]^ (c) Substrate scope of carboxylating enoyl thioester reductases (ECRs). Three ECRs (RevT from *Streptomyces* sp., CinF from *Streptomyces* sp., and EcrSh from *Streptomyces hygroscopicus*) showed broad substrate scope for α,β‐unsaturated CoA‐thioesters. RevT, CinF, EcrSh afforded moderate to excellent conversion of the presented substrates (40–100 % conversion).^[36]^

Erb and co‐workers have devoted a significant efforts towards developing biocatalytic routes for the carboxylation of CoA esters, especially through the development and exploitation of crotonyl‐CoA carboxylase/reductase (CCR), carboxylating enoyl‐thioester reductases (ECRs) and recently, glycolyl‐CoA carboxylase (GCC). Following their discovery of crotonyl‐CoA carboxylase/reductase (CCR) from *Rhodobacter sphaeroides* in 2007, Erb et al. explored the synthetic utility of CCR as a carboxylation catalyst.^[82]^ Initially, they showed that cell extracts of *R. sphaeroides* incubated with NaHCO_3_, NADPH, and acetyl CoA, the C2 CoA ester, acetyl‐CoA **44** was converted into the C5‐carboxylate ethylmaonyl‐CoA **46 a**, in multistep transformations involving a carboxylation step, affording almost full conversion within 1 h of incubation (Scheme 8a).^[82]^

Crotonyl‐coA carboxylase/reductase (CCR) was identified as the carboxylating enzyme, and shown to catalyze the ATP‐free reductive carboxylation of crotonyl‐CoA to ethylmalonyl‐CoA with NADPH as reductant (Scheme 8b).^[82]^ The heterologous expression of the *ccr* gene in *E. coli* allowed the isolation of the carboxylase/reductase CCR, which was applied as the carboxylation catalyst to convert crotonyl‐CoA **46**+CO_2_ to ethylmalonyl‐CoA **46 a**. CCR also showed tolerance towards acryloyl‐CoA.^[35]^

Erb and co‐workers also investigated carboxylating enoyl‐thioester reductases (ECRs) for the carboxylation of a wide range of α,β‐unsaturated CoA‐thioesters.^[36]^ Nine ECRs were assessed against a structurally diverse substrate library (**46**–**59**). Whereas all nine ECRs carboxylated C4 and C5‐enoyl‐CoAs, three of the enzymes, RevT and CinF from *Streptomyces* sp. and EcrSh from *Streptomyces hygroscopicus*, showed a remarkably broad carboxylation substrate scope, affording the carboxylated products **46 a**, **47 a**, and **49 a** in moderate to excellent conversion (up to >99 % conversion; Scheme 8c).^[36]^ They also established that these enzyme families are evolvable by pinpointing active site residues controlling substrate specificity. Thus, rationally designed variants exploring substitutions of these residues improved catalytic efficiency towards, for example, bulkier substrates, such as **48** and **55**.^[36]^

Furthermore, Erb's group successfully improved the weak promiscuous carboxylation activity of a medium‐chain dehydrogenase/reductase (MDR) by protein engineering. Using propionyl‐CoA synthase from *Erythrobacter* sp. NAP1 and acrylyl‐CoA reductase from *Nitrosopumilus maritimus* as template, they generated a rationally designed single point mutant with improved carboxylation efficiency, comparable to natural carboxylases.^[83]^ A notable feature of CCR, ECRs and the engineered promiscuous MDR‐carboxylase is that they do not require ATP. The required nicotinamide cofactor can easily be supplied at scale using well established NAD(P)H cofactor recycling,^[84]^ making preparative scale reactions feasible.

Finally, the group has recently reported the development of glycoyl‐CoA carboxylase (GCC) using weak promiscuous carboxylation activity of biotin‐dependent propionyl‐CoA carboxylases from *Methylorubrum extorquens* (*Me*PCC) towards the carboxylation of glycoyl‐CoA to tartronyl‐CoA (Scheme 8d) as a starting template for protein engineering.^[37]^ Using rational and directed evolution coupled with ultrahigh throughput microfluidic screening, weak carboxylation (catalytic) efficiency of glycoyl‐CoA of *Me*PCC was improved by up 900‐fold to yield two very active variants, GCC M4 and M5, which also remedied the problematic/undesirable ATP hydrolysis associated with the wild type MePCC.^[37]^

## 4 Strategies to Improve Carboxylation Yields

### 4.1 Use of elevated CO_2_ levels as co‐substrate

Several groups have explored the use of elevated concentrations of bicarbonate (HCO_3_^−^, as CO_2_ source) to shift the (de)carboxylation equilibrium towards the carboxylation direction. For example, Wieser et al. investigated the effect of varying the concentration and the type of HCO_3_^−^/CO_3_^2−^ salts as CO_2_ source towards improving carboxylation conversions for P2CDC‐catalyzed reactions.^[65]^ They observed that carboxylation conversion improves with increasing HCO_3_^−^ concentration, attaining peak conversion level at saturating HCO_3_^−^ concentration (>2.5 M). In contrast, CO_3_^2−^ salts, such Li_2_CO_3_, Na_2_CO_3_, and K_2_CO_3_, were not effective as carboxylation agents (Table 1).

**Table 1:** Enzymatic carboxylation utilizing different bicarbonate salts to supply CO_2_ to (de)carboxylase enzymes.

Buffer	Carboxylation conversions [%]^[a]^		
	P2CDC^[a]^	2,6‐DHBD_*Rs* ^[b]^	PAD_*Mc* ^[b]^
(CH_3_)_4_NHCO_3_	–	38	12
KHCO_3_	82	36	30
NH_4_HCO_3_	77	34	33
CsHCO_3_	–	26	28
NaHCO_3_	66	26	22
choline‐HCO_3_	–	24	28
BaCO_3_	14	–	–
CaCO_3_	13	–	–
Li_2_CO_3_	<1	6	5
Na_2_CO_3_	<1	–	–
K_2_CO_3_	<1	–	–

[a] Data taken from ref. [64, 65]. [b] Data taken from ref. [83, 85]. (−) indicates no data.

In a more recent study, Wuensch et al. corroborated the findings of Wieser et al., establishing that for decarboxylase‐catalyzed carboxylation reactions, conversion is affected by the type of bicarbonate salt as well as its concentration. For example, 2,6‐dihydroxybenzoic acid decarboxylase from *Rhizobium sp*. (2,6‐DHBD_*Rs*) and *Mycobacterium colombiense* (PAD_*Mc*) showed clear preference for KHCO_3_ and bicarbonate salts of ammonia, with peak conversions achieved at saturating [HCO_3_^−^], whereas aminoguanidine bicarbonate was a poor co‐substrate.^[85]^ Consequently, for most decarboxylase‐catalyzed carboxylation biotransformation reactions, HCO_3_^−^ is supplied at (near) saturating concentrations (typically 1–3 M HCO_3_^−^).

Wuensch et al. suggest that there is a correlation between the performance of the bicarbonate salt and the Hofmeister ranking. The latter ranks cations and anions based on their ability to salt‐out proteins,^[86]^ showing the kosmotropic (stabilizing) and chaotropic (destabilizing) effects of an ion. In general, the observed conversions attained by 2,6‐DHBD_*Rs* conformed to the cation Hofmeister series with kosmotropic ions such as tetraethylammonium bicarbonate affording higher conversions (37 %), whereas chaotrophic ions such as Li^+^ and guanidinium providing significantly lower conversions (ca. 6 % and 0 % respectively). A similar trend was observed when PAD_*Mc* was employed as the carboxylation catalyst. It is also possible that water solubility of these salts at the reaction pH as well as the slow interconversion of the molecular species, CO_3_^2−^, HCO_3_^−^, CO_2(aq)_ may contribute to the performance of the salts as carboxylating agents.

It has been highlighted that carboxylation methods employing gaseous CO_2_ as the carboxylating agent, instead of large excess of bicarbonate, can eliminate the need to use wasteful amount of bicarbonate salts.^[87]^ In addition, carboxylation using pressurized CO_2_ may simplify downstream reaction work‐up and product isolation. Until recently, examples demonstrating the utilization of gaseous CO_2_ for biocatalytic carboxylation have only been successful when used in combination with elevated concentrations of HCO_3_^−^.[^88^, ^89^, ^90^] However, Faber and co‐workers recently applied pressurized CO_2_ as the sole carboxylating agent for direct *o*‐carboxylation of resorcinol catalyzed by 2,3‐DHBD_*Ao* and SAD_*Tm*. They found that the H_2_CO_3_ generated from dissolved CO_2_ in the reaction buffer causes significant acidification, hence inactivating the biocatalyst. To address this, they optimized the reaction buffering system to alleviate the pH change therefore allowing carboxylation conversion of up to 68 % with pressurized CO_2_ (∼30–40 bar) as the sole carboxylating agent.^[87]^

### 4.2 Carboxylation under supercritical CO_2_ conditions

Supercritical fluids are emerging as important media for enzymatic transformations.^[91]^ Among other advantages, supercritical fluids can allow reaction conditions to be adapted by manipulating pressure and/or temperature, which can improve the solubility of substrates and products. This is particularly attractive for enzymatic carboxylation; the use of supercritical CO_2_ (scCO_2_) as carboxylation reaction medium drastically improves CO_2_ availability and thus potentially carboxylation yield. In addition, CO_2_ has a low critical temperature of 304 K and a critical pressure of 72.9 atmospheres,^[90]^ allowing supercritical state to be reached under ambient conditions suitable for enzymatic transformation.

Given these advantages, the potential for scCO_2_ as a medium for enzymatic carboxylation has been investigated. Matsuda et al. applied whole cells of *B. megaterium* (PYR2910) containing P2DC as the carboxylation catalyst for enzymatic carboxylation of pyrrole in a scCO_2_/H_2_O biphasic system.^[90]^ The yield of the carboxylation reaction was much higher at pressures above the critical pressure of CO_2_ (7.6 MPa) (Table 2), this was also trialed with immobilized cells.^[67]^ Similarly, 2,6‐dihydroxybenzoate decarboxylase from *Pandoraea* sp. 12B‐2 (2,6‐DHBD_*Ps*) has also been tried in scCO_2_, applying the enzyme as whole‐cell preparation. In this instance, 30 mM 2,6‐dihydroxybenzoate was formed from 100 mM 1,3‐dihydroxybenzene (30 % conversion), a slightly lower yield when compared to conversion obtained (45 %) with reaction performed with 3 M KHCO_3_ as a CO_2_ source.^[88]^

**Table 2:** Enzymatic conversion of pyrrole into pyrrole‐2‐carboxylate in scCO_2_.

Pressure [MPa]	Vol.^[a]^ [mL]	pH	*t* [h]	Conversion [%]
0.1 (atmospheric)	0.5	5.5	1	7
0.1 (atmospheric)	0.5	5.5	3	6
10 (supercritical)	0.5	5.5	1	54
10 (supercritical)	0.5	5.5	3	55
10 (supercritical)	0.0	5.5	3	0
10 (supercritical)	1.0	5.5	3	59
10 (supercritical)	0.5	7.0	1	59

[a] Volume of *B. megaterium* cells. *T*=40 °C. Data take from ref. [88, 90].

One important factor to be considered is biocatalyst stability under supercritical conditions. Enzyme inactivation may occur and in turn may result in poor carboxylation yield under scCO_2_. For example, Mn^2+^‐dependent phenylphosphate carboxylase from *T. aromatica* afforded significantly lower conversion under scCO_2_ conditions (5 % conversion)^[56]^ when compared with reaction performed at 100 mM NaHCO_3_ (90 % conversion).^[55]^

Optimization of reaction conditions to enhance catalyst stability and allow scCO_2_ exploitation as a suitable medium for enzymatic carboxylation are key. One study shows that pyruvate decarboxylase loses 80 % of its decarboxylation activity following treatment with pressurized CO_2_ at 60 bar,^[92]^ with enzyme inactivation mitigated through the use of excipients such as glycerol and trehalose, and immobilization on an ion exchange polymer. These interventions led to a dramatic improvement in the biocatalyst stability.

### 4.3 Effect of pH

Reaction pH for enzymatic carboxylation reactions can influence the availability of CO_2_ in the reaction buffer, as a pH‐dependent equilibrium exists between the different dissociation species (H_2_CO_3_, CO_2_(aq), HCO_3_^−^ and CO_3_^2−^). Given that different (de)carboxylases preferentially utilize CO_2_(aq) or its hydrated for HCO_3_^−^ as co‐substrate, identifying and maintaining optimal reaction pH that enhances availability of the desired molecular species is important especially in a closed reaction vessel/system. The reaction pH can also alter the stability of the biocatalysts and other reaction components such as the substrate and the product, in turn affecting the performance of the whole system.

Wuensch et al. gauged the effect of pH on PAD‐catalyzed carboxylation reactions, revealing increasing conversions at between pH 6.5 and 9.5, while a DHBD‐catalyzed carboxylation biotransformation showed a pH optimum between 7.5 and 8.5.^[85]^ At higher pH values, the promiscuous hydration activity mediated by PADs was enhanced, leading to the formation of hydration product as the major product. Miyazaki had also previously found that higher conversion values were achieved with increasing pH, for a PyDC‐catalyzed carboxylation reaction.^[33]^ When the reaction pH was varied between pH 8.5 and 11.5, they found that the carboxylation conversion peaked at pH 11; which can be attributed to the higher rate of hydrolysis of α‐lactoylthiamin at higher pH, typical of thiamine dependent‐systems.^[33]^ In contrast, other decarboxylases have been shown to exhibit optimal (de)carboxylation activity at slightly acidic pH values.^[34]^ It is however difficult to maintain the optimal reaction pH throughout the duration of the reaction, especially under conditions required to favor carboxylation (e. g., at saturating bicarbonate concentrations and/or pressurized CO_2_). Identifying appropriate aqueous reaction buffers represents a successful approach to cushion the effect of pH changes.[^34^, ^87^]

### 4.4 Use of organic cosolvent

Organic co‐solvents have the ability to modify water activity, leading to changes in kinetic and thermodynamic behavior of enzymatic reactions.[^93^, ^94^] Hence, the use of organic co‐solvents can potentially affect the biocatalytic carboxylation reaction rate and the (de)carboxylation equilibrium. Nagasawa and co‐workers examined the effect of several organic co‐solvents revealing that addition of 10–20 % (**v**/**v**) acetone increased the rate of enzymatic carboxylation towards the formation of 2,6‐dihydroxybenzoate catalyzed by 2,6‐DHBD_*Ps*. However, the effect on conversion yields following longer incubation was not significant.^[88]^ Wuensch et al. found that the efficiency of 2,6‐DHBD‐catalyzed *o*‐carboxylation was increased by up to ∼50 % with the addition of 20 % (v/v) of water‐miscible co‐solvents and polyethylene glycols.^[85]^ In particular, DMF, 1,2‐dimethoxyethane, acetone, acetonitrile, **N**‐methyl‐2‐pyrrolidone and 1,4‐dioxane improved conversion values. However, β‐carboxylation biocatalysts appear to be less tolerant to organic co‐solvents investigated.^[85]^ Thus, the use of organic co‐solvents can increase the efficiency of biocatalytic carboxylation reaction, however the compatibility of different co‐solvents has to be determined for the decarboxylase employed.

### 4.5 In situ CO_2_ capture for carboxylation

**Scheme 9:**
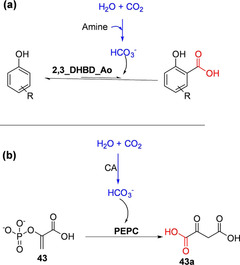
In situ CO_2_ capture and utilization for carboxylation. (a) Linking amine‐mediated CO_2_ capture to carboxylation reaction catalyzed by 2,3‐dihydroxybenzoic acid decarboxylase from *Aspergillus oryzae* (2,3_DHBD_*Ao*).^[97]^ (b) Linking in situ capture of CO_2_ mediated by carbonic anhydrase and its utilization for the carboxylation of phosphoenoylpyruvate (PEP) catalyzed by PEP carboxylase.[^80^, ^81^]

Amine based technology for the capture of CO_2_ is increasingly receiving attention.[^95^, ^96^] Pesci et al. exploited the reversible reactivity of amines with CO_2_ to capture CO_2_ in situ for carboxylation in a biotransformation vessel (Scheme 9a).^[97]^ They linked an in situ amine‐mediated CO_2_ capture to a decarboxylase‐catalyzed carboxylation reaction and monitored the carboxylation efficiency with this system vis‐a‐vis that lacking the amine‐mediated CO_2_ capture component.^[97]^ Biotransformation reactions were performed under high pressure, and a typical reaction mixture contained 2,3‐DHBD_*Ao* as lyophilized *E. coli* cells, buffered amine solution and substrate (catechol), while CO_2_ was slowly supplied up to a pressure of 50 bar. Carboxylation of catechol performed in 1 M KHCO_3_ aqueous solution was compared to reaction performed in 1 M triethylamine (TE)/CO_2_ solution. They observed that both reaction rate and conversion values were higher with the TE/CO_2_ reaction medium (ca. five‐ and two‐fold, respectively). Although a number of primary, secondary and tertiary amines produced similar improvements as the TE/CO_2_, these conditions appear to be specific to catechol, as the same system did not result in improvements in conversion when applied to the carboxylation of other aromatic compounds.^[97]^ This may be due to enzyme inhibition/inactivation at such high concentration of TE, thus less reactive substrates are less likely to be efficiently carboxylated.

In a similar study utilizing triethanolamine (TEA)‐based CO_2_ at a significantly higher concentration (3 M), and employing a CO_2_ fine bubble gassing technique, Ohde et al. achieved subtle improvements in conversion with the same enzyme system. They attributed this improvement (16.1 % to 25.7 %) to the higher concentration of amine mediator (1 M vs 3 M), which in turn allowed CO_2_ to be supplied as ‘fine bubbles’.^[98]^

In another study, the carboxylation yield was shown to improve with the TEA‐mediated CO_2_ in situ delivery linked to a decarboxylase(2,6‐DHBD_**Rs**)‐catalyzed carboxylation of resorcinol, when this system was coupled to a downstream in situ chemical carboxylate precipitation.^[99]^ A quaternary ammonium salt was employed as the precipitant as has been previously demonstrated by Ren et al.^[100]^ However, the efficiency of the system reported by Ohde et al. depends on the type and concentration of the amine mediator, the downstream derivation agent, and reaction conditions.^[98]^

Other investigators have explored the use of carbonic anhydrase (CA) for CO_2_ capture and delivery for enzymatic carboxylation in one‐pot system. CA accelerates the interconversion of CO_2_ and water into bicarbonate, thus ensuring a continuous supply of the co‐substrate for carboxylation. Incorporation of CA to (de)carboxylase‐catalyzed carboxylation reaction to improve the efficiency of biocatalytic carboxylation has been investigated.

Faber and co‐workers explored the carbonic anhydrase (CA)‐mediated CO_2_/(bi)carbonate equilibration process to enhance carboxylation yield, but with no success.^[61]^ It is possible that CA was inhibited by the aromatic substrates/other reaction components/conditions in their system or that CO_2_/bicarbonate equilibrium was not rate limiting. In contrast, Del Prete et al. successfully constructed an in vitro nonlinear one‐pot enzymatic cascade combining a recombinant phosphoenolpyruvate carboxylase (PEPC) and a bacterial carbonic anhydrase, which was reported to improve the efficiency of PEPC‐catalyzed carboxylation of PEP **43** to **43 a** (Scheme 9b).^[81]^ Hwang et al. immobilized PEPC and CA in microbead compartments to develop a stabilized PEPC‐CA carboxylation nonlinear cascade that improved the efficiency of the system and recyclability of the catalysts.^[101]^

In general, despite the potential of the in situ CO_2_ capture and delivery systems, mediated by amines or CAs for enzymatic carboxylation, only subtle improvements in the carboxylation efficiency have been demonstrated so far. This suggests the CO_2_/bicarbonate equilibrium may not be the rate limiting factor in the carboxylation reactions investigated or that the optimal reaction conditions for these orthogonally connected cascade reactions are yet to be identified.

## 5 Biocatalytic CO_2_‐Fixation Cascades for Preparation of Carboxylate Derivatives

Decarboxylases have been frequently employed as selective and beneficial defunctionalization catalysts in enzymatic and chemoenzymatic synthetic cascades.[^102^, ^103^, ^104^, ^105^, ^106^] Even more appealing is the prospect of exploiting reversible decarboxylases as C−H functionalization tools to generate carboxylate derivatives from simple precursors through cascade biocatalysis. This prospect has proven practically challenging, given the inherent thermodynamic constraint associated with the carboxylation step. In recent years, linear enzymatic and chemo‐enzymatic synthetic CO_2_‐fixation cascades have been designed to simultaneously alleviate the thermodynamic ‘inertia’ of CO_2_ fixation and to achieve an economically viable synthetic purpose. Typically, the (de)carboxylase‐catalyzed carboxylation reaction is linked to at least a second transformation step. The latter derivatizes the carboxylation product, often through an irreversible reaction or an energetically favorable downstream reaction, removing it from the (de)carboxylation equilibrium. This tandem process often explores the reactivity of the carboxylic acid group or other functional groups proximal to the carboxylic acid moiety such that the derivatized/downstream product is no longer reactive with the decarboxylase. Artificial synthetic CO_2_‐fixation pathways developed in these ways are often inspired by and mimic natural microbial synthetic pathways; and often recruit suitable biocatalysts from diverse sources/pathways and in some cases abiotic catalysts or reagents.

### 5.1 Chemoenzymatic synthetic derivatization approaches

**Scheme 10:**
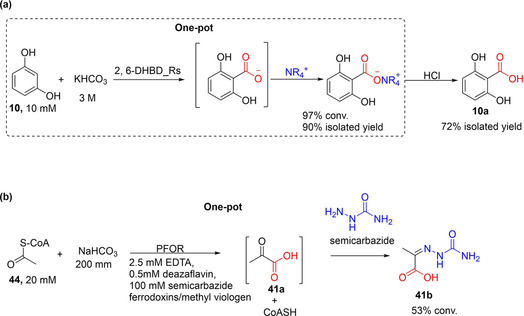
Enzyme‐catalyzed CO_2_ fixation linked to chemical derivatization of the carboxylate product. (a) A strategy applying quaternary ammonium salt to shift the reaction equilibrium towards 1,6‐DHBD_*Rs*‐catalyzed carboxylation of resorcinol. The carboxylation step is linked to the precipitation of the carboxylate in one pot. The product was recovered by acid hydrolysis. 1,6‐DHBD_*Rs*=1,6‐dihydroxybenzoic acid decarboxylase.^[100]^ (b) Pyruvate:ferredoxin oxidoreductase (PFOR)‐catalyzed CO_2_ fixation linked with derivatization using semicarbazide.^[107]^

Ren et al. exploited the reactivity of the carboxylic acid group towards quaternary ammonium salts.^[100]^ The latter when added to a decarboxylase‐catalyzed carboxylation biotransformation, reacts with the carboxylic acid moiety of the carboxylation product to form an insoluble salt, thereby removing it from the equilibrium (Scheme 10a). The resulting salt can subsequently be hydrolyzed using a strong acid to regenerate the carboxylic acid. Using the carboxylation of resorcinol **10** catalyzed by 2,6‐DHBD_*Rs* as the model reaction, eight quaternary ammonium salts and tetrabutylphosphonium were screened as precipitating agents. Tetrabutylammonium bromide performed as the superior precipitating agent under the conditions investigated. The decarboxylase‐catalyzed carboxylation reactions containing 2 and 5 equivalents of tetrabutylammonium bromide afforded 80 and 97 % conversions, respectively, representing significant improvements when compared to conversion obtained (37 %) with a control reaction lacking the precipitant. Initial reaction rates were comparable for reaction containing or lacking the precipitant, indicating that enzyme velocity is unaffected. However, while the reaction containing the precipitants progressed to near completion after 24 h, the yield with the control reaction reached a plateau. Product recovery was enabled by acid hydrolysis, hence 2,6‐dihydroxybenzoic acid was isolated in yield of up 72 %. Carboxylation of catechol was also accomplished using this approach, in this case dodecyldimethylbenzylammonium chloride or tetradecyldimethylbenzylammonium chloride was used as the precipitant.^[100]^ In addition, the precipitating quaternary ammonium compound is recoverable through extraction with an appropriate organic solvent (e. g., chloroform). Zhang et al. has recently applied this strategy to improve carboxylation conversion values employing 2,3‐DHBD_*Fo* as the carboxylation catalyst,^[64]^ and Ohde et al. showed that this in situ crystallization approach improved performance of their amine‐mediated CO_2_ capture‐carboxylation system under optimized conditions.^[99]^

The in situ carboxylate precipitation of Ren et al. may be adaptable for use as a generic approach to improve carboxylation yield, although an appropriate precipitating agent may need to be identified for each product type. Crucially, it is yet to be established whether this approach can be extended to other (de)carboxylases as this system has only thus far been demonstrated for related DHBDs.

Other chemical derivatizing agents have been explored for enzymatically produced carboxylates as a strategy to improve carboxylation conversion. For example Witt et al. recently devised a chemical derivatization strategy to drive the carboxylation of acetyl CoA **44** by using pyruvate:ferredoxin oxidoreductase (PFOR) as the carboxylation catalyst.^[107]^ PFOR catalyzes the interconversion of **44** and CO_2_ to pyruvate **41 a**, however the carboxylation (oxidative) reaction is severely disfavored. Given that the carboxylation product **41 a** contains an α‐keto group which can react with semicarbazide to form the corresponding semicarbazone condensation product **41 b**; Witt et al. hence targeted the reactivity of the α‐keto group of the formed pyruvate by adding semicarbazide as a derivatizing agent in the carboxylation biotransformation (Scheme 10b). Using this strategy and employing non‐native ferredoxin or methylviologen as cofactor, the derivatized carboxylation product (pyruvate semicarbazone, **41 b**) was detected in conversion of up to 53 %.^[107]^ This approach demonstrates that enzymatic CO_2_ fixation to form carbonyl group‐containing products, such as α‐keto acids, can be linked to semicarbazide condensation to generate biologically active semicarbazones. Alternatively, PFOR‐catalyzed carboxylation can be linked to other enzymatic transformation targeting the α‐keto group, for example through asymmetric transamination/reductive amination or carbonyl reduction to yield the corresponding optically pure amino acids or hydroxy acids, respectively.

### 5.2 Synthetic enzymatic CO_2_‐fixation cascades for derivatization and valorization

#### 5.2.1 Conversion of aldehydes into hydroxy/amino acids via α‐keto acids

**Scheme 11:**
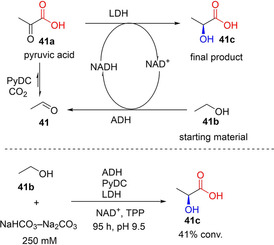
Enzymatic CO_2_‐fixation cascade for the conversion of ethanol and CO_2_ into lactic acid through pyruvate decarboxylase (PyDC)‐catalyzed carboxylation.^[108]^

Tong et al. developed a multienzyme system for the conversion of CO_2_ and acetaldehyde **41** to **41 a** which is subsequently reduced to l‐lactic acid **41 c**. The synthetic concept employs ethanol **41 b** as a ‘smart’ substrate; alcohol dehydrogenase(ADH)‐catalyzed oxidation of **41 b** generates the carboxylation substrate, **41** as well as the reductant NADH (Scheme 11).

The multistep process involves (i) an NAD‐dependent oxidation of **41 b** to generate **41** and NADH, catalyzed by alcohol dehydrogenase; (ii) carboxylation of **41** by pyruvate decarboxylase (PyDC) using carbonate/bicarbonate buffer as CO_2_ source, forming **41 a**; (iii) NADH‐dependent enantioselective reduction of **41 a** to generate **41 c**, and NAD^+^ achieving redox neutral NAD(H) regeneration (Scheme 11). Using this approach in a batch process and with continuous feeding of **41 b**, 41 % of ethanol was converted into **41 c**, albeit after 4 days.

The concept of using ethanol as the smart substrate is elegant and economically attractive. However, a simpler two‐step linear process linking acetaldehyde carboxylation and pyruvate reduction, and employing alternative NADH cofactor recycling systems (e. g., glucose dehydrogenase‐based systems) may address the poor cofactor recycling turnover observed with the system described by Tong et al.

**Scheme 12:**
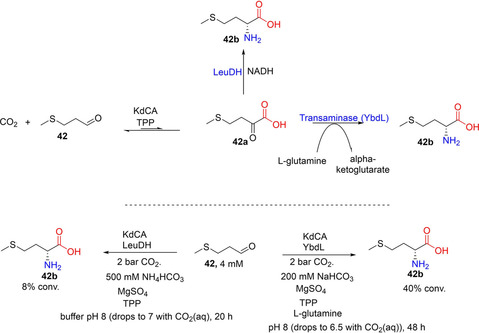
One‐pot enzymatic cascade for the conversion of aldehyde into amino acid involving CO_2_‐fixation step catalyzed by TPP‐dependent decarboxylase (KdCA) and a reductive amination step catalyzed either by a transaminase (YbdL) or an amine dehydrogenase (LeuDH).^[34]^ TPP=thiamine pyrophosphate, LeuDH=leucine dehydrogenase.

Skerra and co‐workers have also exploited an enzymatic cascade to demonstrate the application of a TPP‐dependent decarboxylase KdcA for CO_2_ fixation. By linking KdCA‐catalyzed CO_2_ fixation of an aldehyde (e. g., methional, **42**) to reductive amination of the formed keto‐acid **42 a**, catalyzed by a transaminase (ydL) or an amino acid dehydrogenase (LeuDH or PheDH), L‐methionine **42 b** and related amino acids were accessed (Scheme 12). Conversion of up to 40 % was attained after 48 h incubation at 2 bar CO_2_ pressure and 200 mM NaHCO_3_ (or 500 mM NH_4_HCO_3_).^[34]^ A notable issue concerns the incompatibility of reaction conditions, especially with respect to identifying buffer systems that ensure compatible reaction pH for the decarboxylase and the amination steps. In addition, incorporation of NAD(P)H co‐factor recycling system for the dehydrogenase step can improve applicability of the system, while the product profile may further be extended to *N*‐alkyl amino acids by employing *N*‐alkyl amino acid dehydrogenases^[109]^ as the amination catalysts.

#### 5.2.2 Conversion of nonactivated aromatic compounds into alcohols, amines, and amides

**Scheme 13:**
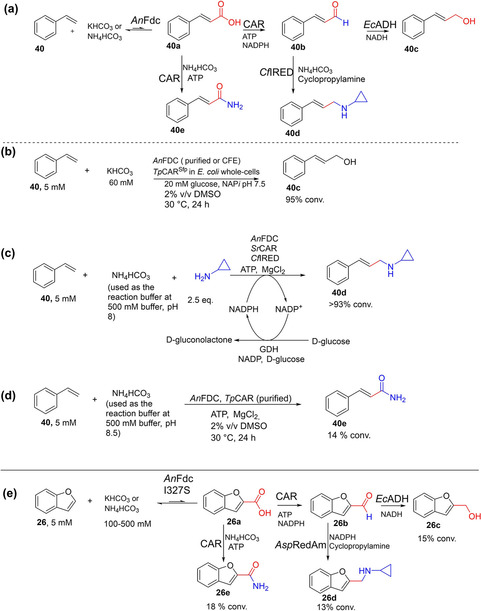
Enzymatic CO_2_‐fixation cascades for the conversion of aromatic compounds into alcohols and amines.^[43]^ (a) CO_2_‐fixation cascades enabling the conversion of styrene into the corresponding allylic alcohol and amine.^[43]^ (b) One‐pot three‐step enzymatic CO_2_‐fixation cascade for the conversion of styrene into cinnamyl alcohol, involving a sequence of Fdc‐catalyzed carboxylation, CAR‐catalyzed carboxylate reduction, and carbonyl reduction catalyzed by endogenous alcohol dehydrogenase.^[43]^ (c) One‐pot three‐step artificial CO_2_‐fixation cascade involving a sequence of Fdc‐catalyzed carboxylation, CAR‐catalyzed carboxylate reduction and reductive aminase(RedAm)‐catalyzed reductive amination, enabling the conversion of styrene into the corresponding secondary amines; reaction contained (NH_4_)HCO_3_ as CO_2_ source and cyclopropylamine as the primary amine source, and incorporated GDH‐based recycling of NADPH.^[43]^ (d) One‐pot artificial CO_2_‐fixation cascade enabling the conversion of styrene into the corresponding cinnamide involving AnFDC‐catalyzed carboxylation linked to CAR‐catalyzed amidation using NH_4_HCO_3_ as source of both CO_2_ and NH_3_. (e) CO_2_‐fixation cascades enabling the conversion of benzofuran into the corresponding alcohol, amine, and amide featuring similar conditions to those for (b), (c), and (d) respectively but employing the engineered heteroaromatic (de)carboxylase *An*FDC I327S as the carboxylation catalyst.^[43]^ *Tp*CAR=*Tsukamurella paurometabola* carboxylic acid reductase; *Sr*CAR=*Segniliparus rugosus* carboxylic acid reductase; *An*Fdc=ferulic acid decarboxylase from *Aspergillus niger*; *Cf*IRED=*Cystobacter ferrugineus* imine reductase; GDH=glucose dehydrogenase.

The versatility and evolvability of prFMN‐dependent fungal ferulic acid decarboxylases (FDCs) provide an opportunity to expand the synthetic scope of biocatalytic carboxylation reactions, for example towards functionalization of nonactivated aromatic compounds. Significant insights into prFMN[^39^, ^70^, ^71^, ^110^, ^111^] and FDC biochemistry[^30^, ^112^] and (de)carboxylation substrate scope^[41]^ supported development of FDC‐based carboxylation routes to enable access to industrially prevalent carboxylate derivatives including aldehyde, alcohols, amides and amines from simple nonactivated aromatic compounds by biocatalytic CO_2_‐fixation cascades.^[43]^ To address the severe equilibrium limitation encountered with the one‐step FDC‐catalyzed carboxylation reaction, the FDC‐mediated CO_2_ fixation^[43]^ was linked to carboxylate reduction, the latter step catalyzed by carboxylic acid reductases (CARs).[^113^, ^114^, ^115^] Further valorization of the process was achieved through functional group interconversion.[^116^, ^117^] The intermediate aldehyde is converted into the corresponding alcohol or amine through carbonyl reduction (catalyzed by endogenous alcohol dehydrogenase) or reductive amination catalyzed by reductive aminases^[118]^ (Scheme 13a–c). Thus, a three‐step cascade involving *An*FDC‐catalyzed carboxylation, CAR‐catalyzed carboxylate reduction and subsequent enzymatic carbonyl reduction/reductive amination, implemented as a one‐pot biotransformation, converted styrene **40** into the corresponding allylic alcohol **40 c** or allylic amine **40 d** via cinnamic acid **40 a** and cinnamaldehyde **40 b** intermediates, respectively (Scheme 13a–c). These systems afforded high conversion values (up to 95 %) to the target products, even at low bicarbonate concentration (<100 mM).^[43]^ This strategy was also extended to the C−H functionalization of heteroaromatic scaffolds such as benzofuran **26** albeit in lower conversions; in this instance (Scheme 13e), an engineered heteroaromatic (de)carboxylase (*An*FDC I327S) was applied as the carboxylation catalyst.^[43]^

Furthermore, the (de)carboxylase‐CAR CO_2_‐fixation system was extended towards the synthesis of amides **40 e** and **26 e** from **40** and **26**, respectively, by linking the recently developed CAR‐catalyzed amidation reaction (NADPH‐free CAR‐catalyzed process)^[119]^ to the FDC‐catalyzed CO_2_ fixation (Scheme 13d,e). Notably, NH_4_HCO_3_ buffer served as source for both CO_2_ and NH_3_ required for the carboxylation and amidation steps, respectively. This process however resulted in lower conversion, generating the corresponding amide in up to 15 % (Scheme 13d,e), perhaps owing to the low efficiency of the CAR‐mediated amidation reaction. Recently, amide bond‐forming biocatalysts have attracted significant attention, and more efficient and substrate promiscuous amide bond‐forming enzymes are emerging.[^120^, ^121^, ^122^] These amidases may be explored as alternative amidation catalysts in the synthesis of amides by CO_2_ fixation.

The enzymatic carboxylation linked to CAR represents potentially a generic approach given the remarkable substrate profile of CAR enzymes[^113^, ^123^, ^124^] and the versatility of aldehyde products as intermediates to access a wide range of other functional groups and conjugation products.

### 5.3 Synthetic in vitro CO_2_ fixation cycles for chemical production

The Calvin‐Benson‐Bassham (CBB) cycle of plants, algae and various autotrophic microorganisms is responsible for more than 90 % of global natural CO_2_ fixation, with the rest converted via alternative CO_2_ fixation pathways. Despite this naturally existing prevalence, the application of CO_2_‐fixing enzymes and pathways for converting CO_2_ into value‐added multi carbon products has been limited in chemical and biotechnological applications. As with natural CO_2_‐fixation processes, a familiar bottleneck is the efficiency of the carboxylating enzyme and attempts to improve abundant natural carboxylases have achieved only limited success. In vitro cascade biocatalysis provides an alternative approach to create completely artificial CO_2_ fixation pathways that are kinetically or thermodynamically favored and can be tuned towards production of economically viable chemicals. To tap into this potential, more efficient carboxylases must be identified to support synthetic CO_2_‐fixation artificial cycles.

Having previously developed and showcased the synthetic potential of enoyl‐CoA carboxylase/reductase (ECR) for a one‐step carboxylation reaction, Erb and co‐workers exploited this carboxylase to develop a novel artificial enzymatic CO_2_‐fixation cycle to a target product. ECR enzymes possess a broad substrate range, are oxygen‐insensitive, and require only the ubiquitous redox cofactor NADPH to catalyze the fixation of CO_2_.

**Scheme 14:**
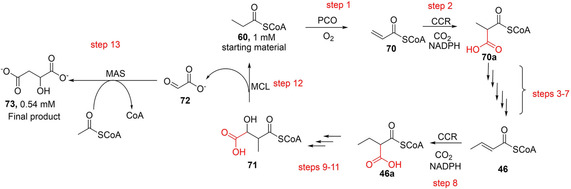
Artificially developed CETCH cycle applied to the synthesis of malate. The multienzyme cascade employs 17 enzymes catalyzing 13 linear enzymatic steps including two reductive carboxylation steps catalyzed by CCR, and additional auxiliary steps for cofactor recycling. A 520 μL reaction was capable of fixing 1080 μM CO_2_ and afforded 540 μM malate in 90 min.^[23]^ PCO=propionyl‐CoA oxidase; CCR=crotonyl‐CoA carboxylase/reductase; MCL=β‐methylmalyl‐CoA lyase; MAS=malate synthase. Propionyl‐CoA: **60**; acrylyl‐CoA: **70**; methylmalonyl‐CoA: **70 a**; crotonyl‐CoA: **46**; ethylmalonyl‐CoA: **46 a**; methylmalyl‐CoA: **71**; glyoxylate:**72**; malate: **73**.

By performing retrobiosynthetic analysis (possibly inspired by the work of Bar‐*Even* et al.^[125]^), Schwander et al. constructed an artificial crotonyl‐CoA/ethylmalonyl‐CoA/hydroxybutyryl‐CoA (CETCH) cycle^[23]^ which was further developed into a fully functional cycle using the typical design/build‐test‐optimize cycles. The CETCH cycle recruits enzymes from a wide range of different biological sources, and the combination of such diverse enzymes into a synthetic pathway posed several challenges. Schwander et al. addressed these problems stepwise, through reaction optimization and protein engineering, improving the process through the different optimized versions of the cycle (CETCH 1–5.4). By version 5.4, the CETCH cycle has been well‐tailored for the synthesis of malate **73** from propionyl‐CoA **60** (Scheme 14). A total of 17 enzymes from nine different organisms were included, with 13 making up the core cycle, and 4 allowing for cofactor regeneration, metabolite proofreading, and rate measurement. Three of the core reactions were created by rational active‐site engineering of existing enzyme scaffolds to catalyze the desired activities. CETCH 5.4 relies solely on the reductive carboxylation of enoyl‐CoA esters by the application of ECRs and displays a CO_2_ fixation rate of 5 nmol min^−1^ mg^−1^ of core cycle proteins. This rate is comparable to reported attempts to measure the CBB cycle in cell extracts, which display reported rates between 1 to 3 nmol min^−1^ mg^−1^.^[23]^ The CETCH pathway represents a successful in vitro reconstitution of a synthetic enzymatic network for the conversion of CO_2_ into organic products.

Further work expanding on the application of the CETCH system is reported by Miller et al.^[126]^ They developed a partially synthetic thylakoid membrane‐based energy‐module (TEM) which can be applied to a range of ATP and NADPH dependent enzymatic systems such as CETCH. The TEM consists of thylakoid membranes derived from spinach (*Spinacia oleracea*) which, when supplemented with ferredoxin, and an external light source, are capable of the light dependent synthesis of ATP and NADPH, with specific activities of 6.5±0.5 mM min^−1^ mg^−1^ chlorophyll (Chl) and 3.41±0.01 μmol min^−1^ mg^−1^ total Chl, respectively.

Initial trials of the light‐driven TEM chemical energy generation system demonstrated its applicability to in vitro CO_2_ fixation, demonstrating a greater than 3 orders of magnitude increase in activity in comparison to other recent attempts to link CO_2_ fixation to isolated thylakoid membrane complexes. When coupled with either the NADPH dependent crotonyl‐coenzyme A (CoA) carboxylase/reductase (CCR), or the ATP dependent propionyl‐CoA carboxylase (PCC), the TEM system displays consistent light‐driven CO_2_ fixation of between 5.1 and 5.2±0.2 μM min^−1^ mg^−1^ Chl.^[126]^

In addition to its ability to power single‐step enzymatic CO_2_‐fixation systems, the TEM construct has been shown to be applicable in driving complete metabolic cycles for the continuous fixation of CO_2_. The combination of 16 enzymes in a modified CETCH cycle, with a glyoxylate/hydroxypyruvate reductase (Ghr) from *E. coli*, allowed for the TEM‐driven production of 156 mM glycolate from 120 mM acceptor molecule.^[126]^ These results demonstrate that the synthetic CETCH cycle can be functionally coupled to the native energy machinery of photosynthesis in a fully integrated fashion using light energy to form multi‐carbon compounds from CO_2_.

**Scheme 15:**
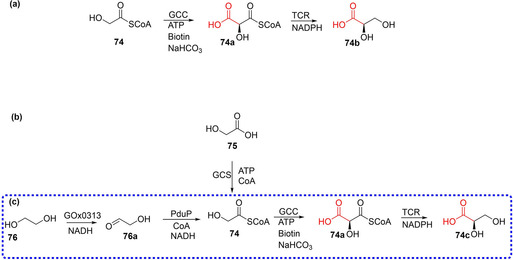
Artificially developed TaCo CO_2_‐fixation cascade and its application in de novo synthesis. (a) TaCo‐based enzymatic cascade enabling conversion of glycolyl‐CoA to glycerate.^[37]^ (b) Conversion of glycolate to glycerate using a TaCo based enzymatic cascade. (c). Conversion of ethylene glycol to glycerate in a 4‐step TaCo‐based enzymatic cascade. Enzymes: GCS=glycolyl‐CoA synthetase; GCC=glycolyl‐CoA carboxylase; TCR=tartronyl‐CoA reductase; GOx0313=alcohol dehydrogenase from *Gluconobacter oxydans*; PduP=aldehyde dehydrogenase from *Rhodopseudomonas palustris* BisB18.

Recently, Scheffen et al. exploited the synthetic utility of their engineered GCC M4 variant to experimentally accomplish a previously designed hypothetical tartronyl‐CoA (TaCo) CO_2_‐fixation pathway.[^37^, ^127^] The TaCo pathway converts **74** (C2 compound) via a carboxylate intermediate **74 a** to glycerate **75 b** (C3) through an enzymatic cascade involving a key CO_2_‐fixation step catalyzed by GCC M4 (Scheme 15a).^[37]^ A more de novo synthetic process was developed starting from glycolate **75** en route to glycerate synthesis (Scheme 15b). **75** can readily be accessed from 2‐phosphoglycolate and propionyl‐CoA through the Calvin‐Benson‐Bassham (CBB) cycle and the artificially developed CECTH respectively.^[37]^ The TaCo pathway also enabled a novel pathway for recycling of polyethylene terephthalate (PET) wastes such as ethylene glycol **76**. By extending the TaCo system with two upstream additional enzymatic steps, ethylene glycol **76** can be converted into glycerate **74 b** (Scheme 15c).^[37]^

## 6 Exploiting prFMN's Catalytic Capabilities: Expanding the Biocatalytic (De)carboxylation Toolbox

### 6.1 Development of broad spectrum hetero(aromatic) carboxylic acid (de)carboxylases

**Scheme 16:**
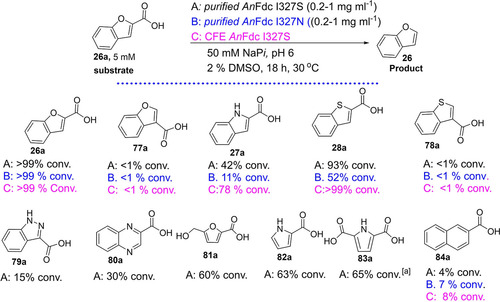
Engineered ferulic acid decarboxylase for (de)carboxylation of (heteroaromatic) carboxylates. Substrate profiling study of *An*Fdc variants I327S/N using purified enzyme preparation of I327S (denoted as A) or I327N (denoted as B), as well as the cell‐free extract of I327S (denoted as C).^[43]^ *An*Fdc is a prenylated flavin (prFMN)‐dependent reversible decarboxylase from *Aspergillus niger*.

To expand the (hetero)aromatic carboxylation toolbox, a structure‐guided semi‐rational protein engineering approach was used to evolve the well‐studied prFMN‐dependent *Aspergillus niger* Fdc (*An*Fdc).^[43]^ This work aimed to develop broad spectrum heteroaromatic (de)carboxylases that can provide access to structurally diverse oxygen‐, nitrogen‐ and sulfur‐containing heteroaromatic carboxylates and their derivatives. First, using the decarboxylation of cinnamic acid as a model reaction, Aleku et al. screened a library of (hetero)aromatic acids to identify heteroaromatic carboxylates that competitively inhibited the native reactions. In this way, they identified benzofuran, indole, benzothiophene carboxylates **26 a**–**28 a** as well as naphthoic acid **81 a** as competitive ‘inhibitors’/alternate substrates of *An*Fdc. To gain structural insights into the binding behavior of these compounds, crystallographic studies were performed. Analysis of crystal structure complexes of *An*Fdc with (hetero)aromatic acid ‘inhibitors’ versus complexes with the native cinnamic acid substrate highlights active site residues constituting steric hindrance and inhibiting the productive binding of the aromatic acid substrates. These residues were subjected to site‐saturation mutagenesis, leading to the identification variants at M238 and I327 showing up to 150‐fold improvement over the weak activity observed for the wild‐type enzyme. Substrate profiling of the I327S/N variants revealed remarkably broad substrate scope towards the decarboxylation of *O*‐, *N*‐, and *S*‐containing heteroaromatic carboxylic acids, **26 a**–**28 a** and **79 a**–**83 a** (Scheme 16).^[43]^

The *An*Fdc I327S/N variants are a valuable addition to the heteroaromatic carboxylation toolbox and extend considerably the potential synthetic scope of biocatalytic carboxylation to access a wide range of heteroaromatic carboxylates. A proof of concept application of the *An*Fdc I327S variant was demonstrated for C−H functionalization of benzofuran through a novel cascade reaction.^[43]^ Crucially, this work highlights the evolvability of the Fdc enzyme class, which can further be exploited to extend the synthetic scope and improve catalytic efficiency.

### 6.2 Towards biocatalytic regioselective C−H carboxylation of unactivated arenes

The regioselective carboxylation of benzene and naphthalene remains a challenging reaction given the entropic requirement of this transformation. Kanan and co‐workers recently developed Cs_2_CO_3_^−^ mediated CO_2_ fixation process that afforded the C−H carboxylation of benzene. However, this process required molten conditions and generated a mixture of benzoate, phthalates, and tri‐ and tetracarboxylates.^[11]^ (De)carboxylase‐based CO_2_‐fixation routes are conceived as alternative green and regioselective methods for the functionalization of these recalcitrant aromatic compounds. However, identifying enzyme candidates capable of catalyzing (de)carboxylation of benzene/naphthalene remains a difficult task.

In recent studies, putative UbiD‐like benzene (de)carboxylase and naphthalene (de)carboxylase have been suggested to be responsible for the weak carboxylase activity observed in cultures capable of degrading benzene and naphthalene respectively.[^128^, ^129^] It has been proposed that the degradation of these compounds occurs via an initial carboxylation reaction, although biochemical characterization and in vitro activity of these enzymes have yet to be demonstrated.

More recently, Aleku et al. identified a single point mutant of *An*Fdc I327N that displays weak decarboxylation activity towards naphthoic acid **84 a** affording up to 8 % conversion into naphthalene **84** and establishing it as the first enzyme to decarboxylate naphthoic acid (Scheme 16a).^[43]^ It is possible that this weak naphthalene (de)carboxylating activity may be further improved and the scope be extended towards benzene/benzoic acids and other polycyclic aromatic hydrocarbons with subsequent rounds of evolution. PrFMN‐mediated (de)carboxylation of naphthoic/benzoic acid is of interest from both mechanistic and application viewpoints and highlights the enormous catalytic potential of these enzymes.[^40^, ^70^, ^130^, ^131^, ^132^, ^133^]

### 6.3 Towards biocatalytic regioselective C−H carboxylation of aliphatic hydrocarbons

**Scheme 17:**
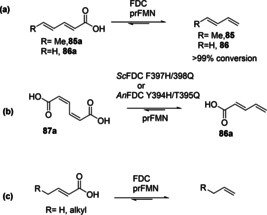
Synthetic scope of prFMN dependent ferulic acid decarboxylases (FDCs) for (de)carboxylation of aliphatic carboxylic acids.[^41^, ^73^, ^136^]

In view of the industrial importance of aliphatic carboxylates and their derivatives, site selective functionalization of aliphatic hydrocarbons at a sp^2^ or sp^3^ carbon through C−H carboxylation is highly attractive. In this regard, prFMN‐dependent FDCs have shown some promise albeit in the decarboxylative direction. For example, FDCs have been shown to catalyze the decarboxylation of conjugated 2,4‐diunsaturated aliphatic monocarboxylic acids (**85 a**, **83 a**),^[41]^ 2,4,6‐tri‐unsaturated acids (e. g., 2,4,6‐octatrienoic acid),^[134]^ aliphatic acids containing isolated α,β‐mono‐unsaturated acids,^[135]^ and 2,6‐dienoic acids (e. g., (*E,E)*‐muconic acid, **87 a**)^[136]^ (Scheme 17).

The substrate tolerance of FDCs and related prFMN‐dependent enzymes towards these aliphatic α,β‐ unsaturated carboxylic acids promises a valuable route to extend biocatalytic CO_2_‐fixation portfolio to the functionalization of aliphatic substrates. However, to date, these enzymes have yet to be exploited as carboxylation catalysts for the functionalization of aliphatic hydrocarbons, perhaps, due to the difficulty with handling of these substrates (e. g., volatility, solubility) and the poor efficiency of these enzymes towards aliphatic substrates. Nonetheless, the evolvability of these enzymes should allow their development towards efficient functionalization of aliphatic hydrocarbons such as aliphatic terminal alkenes through C−H carboxylation. In addition, reaction engineering and optimization of reaction conditions may address issues with substrate handling.

## 7 Conclusion and Outlook

The application of reversible (de)carboxylases as carboxylation catalysts represents a promising biocatalytic route for the incorporation of CO_2_ into organic molecules under benign reaction conditions. With the expanding toolbox of enzymes capable of catalyzing (de)carboxylation of a wide range of structurally diverse molecules, there is a clear opportunity to expand the scope of application and the product profile of biocatalytic carboxylation methods. To build on recent achievements in biocatalytic carboxylation and to enhance the potential of biocatalytic carboxylation processes for industrial exploitation, several aspects of the (de)carboxylase‐catalyzed carboxylation reactions will need to be improved.

The first challenge to be met is the development of truly benign and simple enzymatic carboxylation processes. Most biocatalytic carboxylation reactions were demonstrated at a saturating bicarbonate level or high CO_2_ pressure. The use of pressurized CO_2_ for enzymatic carboxylation renders the process hazardous and requires sophisticated equipment, while carboxylation performed at saturating concentrations of bicarbonate represents a wasteful use of reagent and complicates downstream reaction work‐up. Hence, there is a need to develop simple, safe, and mild enzymatic carboxylation methods that are efficient under low CO_2_ pressure/near stoichiometric concentrations of bicarbonate. In this regard, emerging strategies devised to alleviate the disfavored thermodynamic carboxylation equilibrium are promising. For example, it has been possible to perform reaction at low levels of bicarbonate/CO_2_ supply when the carboxylation step is coupled with chemical/enzymatic derivatization transformations. Promising strategies such as crystallization of the carboxylate product with quaternary ammonium salts, or enzymatic derivatization through biocatalytic carboxylate reduction can further be adapted as generic platforms across different decarboxylases families and for different substrate groups. To enhance the viability of CO_2_ as a building block for industrial exploitation, it is crucial to expand the portfolio of generic strategies available to alleviate the inherent thermodynamic constraint of CO_2_ fixation.

A second challenge is the requirement to significantly expand the substrate scope and product profile of biocatalytic carboxylation. Despite significant addition to the biocatalyst toolbox for carboxylation in the last decade; several substrate groups are yet to be covered. For example, unfunctionalized hydrocarbons as well as nonactivated aromatic compounds whose corresponding carboxylates/derivatives feature significantly as structural components in industrial chemicals, remain difficult to functionalize through enzymatic carboxylation. Directed evolution of existing enzymes as well as biochemical screening of genomic sequences may reveal novel (de)carboxylases with distinct substrate specificity. In addition, functional metagenomic screening supported by appropriate (ultra)high throughput screening and selection techniques[^137^, ^138^] can enhance access to novel decarboxylases, especially for scaffolds that are currently inaccessible through enzymatic carboxylation.

Finally, proof of concept preparative biocatalytic carboxylation reactions simulating conditions often used in industrial processes, such as high substrate loading and incorporating co‐factor recycling systems will greatly enhance the attractiveness of enzymatic carboxylation for industrial exploitation. Recent advances in ATP‐cofactor recycling systems[^139^, ^140^, ^141^] can be explored to improve applicability of ATP‐dependent carboxylation processes. It may be necessary to employ green organic co‐solvents or biphasic systems to improve substrate solubility, prevent product inhibition and enhance the performance of the system. Additionally, improving performance of enzymatic carboxylation maybe achieved using immobilization techniques,^[142]^ and investigating the suitability of batch or flow processes for individual reactions.

## Conflict of interest

The authors declare no conflict of interest.

## Biographical Information

*Dr Godwin Aleku. Godwin completed an M.Sc. in Biotechnology and Enterprise in 2013 at the University of Exeter. He obtained a PhD in chemical biology in 2017, working with Professor Nicholas Turner at the University of Manchester, on the discovery and development of reductive aminases. He was a postdoctoral research associate at the Manchester Institute of Biotechnology between 2017 and 2020, in the group of Professor David Leys. In mid‐2020, he moved to the University of Cambridge, where he is a Leverhulme/Isaac Newton Trust early career fellow in the department of Biochemistry. His current research focuses on the development of a new generation of biocatalysts catalyzing industrially relevant and synthetically challenging organic reactions*.

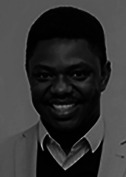

## Biographical Information

*Professor David Leys. David obtained a Ph.D. in Biochemistry/Structural Biology in 2000, at the University of Ghent, Belgium, working on the structure and function of a range of redox enzymes. Following a brief postdoctoral interlude at the University of Edinburgh, David moved to the University of Leicester and was awarded a Royal Society University Research Fellowship in 2003. He relocated to the University of Manchester in 2005, where his research has focused on structural and mechanistic characterization of novel enzymes with particular relevance to biotechnological applications*.